# Supplementary material for: Association of Thoracic Skeletal Muscle Index with Clinical Outcome and Response to Nutritional Interventions in Patients at Risk of Malnutrition—Secondary Analysis of a Randomized Trial
Source: Nutrients. 2023 Feb 5;15(4):817. doi: 10.3390/nu15040817 (PMC9964333; doi:10.3390/nu15040817)
Supplement: Supplementary file 1 [file nutrients-15-00817-s001.zip › nutrients-2176177-supplementary.pdf]

Supplementary Table S1. Association of low SMI at level L3 and clinical outcomes compared

	**high SMI at level L3**	**low SMI at level L3**		**AUC**
	n(%) of Patients with high SMI*	*n*(%) of Patients with low SMI	OR or Coefficient (95% CI)	
			p value, adjusted for age, BMI, nutritional support intervention, contributing center, presence of stroke, COPD, hypertension, diabetes, chronic heart failure	
	***n* = 388**	***n* = 131**		
**Primary endpoint**				
**Adverse clinical outcome within 30 days**	113 (29.1%)	39 (29.8%)	1.1 (0.68, 1.79), *p* = 0.680	0.50
**Short-term endpoints**				
**30-day all-cause mortality**	36 (9.3%)	16 (12.2%)	1.61 (0.79, 3.28), *p* = 0.192	0.53
**Rehospitalisation within 30 days**	48 (12.4%)	13 (9.9%)	0.77 (0.38, 1.56), *p* = 0.470	0.48
**Mean length of stay, days (SD)**	10.1 (7.1)	10.1 (7.1)	-0.02 (-2.55, 1.49), *p* = 0.974	-
**Decline Barthel index score (points)) after 30 days**	59 (15.2%)	20(15.3%)	1.09 (0.58, 2.05), *p* = 0.771	0.50
**Long-term endpoints**				
**180-day all-cause mortality**	126 (32.5%)	40 (30.5%)	0.98 (0.54, 1.46) *p* = 0.660	0.49

Abbreviations: SD denotes standard deviation; OR denotes odds ratio; CI denotes confidence interval; AUC denotes area under the curve

Supplementary Table S2. Association of low SMI and clinical outcomes in different subgroups

	high SMI	low SMI		AUC	Male	Female	NRS ≥ 4	NRS < 4	Age ≥ 80 years	Age < 80 years
	n(%) of patients with high SMI	*n*(%) of patients with low SMI	OR or *Coefficient (95% CI)		OR or Coefficient (95% CI)	OR or Coefficient (95% CI)	OR or Coefficient (95% CI)	OR or Coefficient (95% CI)	OR or Coefficient (95% CI)	OR or Coefficient (95% CI)
			p value, adjusted		*p* value, adjusted	*p* value, adjusted	*p* value, adjusted	*p* value, adjusted	*p* value, adjusted	*p* value, adjusted
	***n* = 496**	***n* = 167**								
**Primary endpoint**										
**Adverse clinical outcome within 30 days**	135 (27.2%)	52 (31.1%)	1.6 (1.03 to 2.5), *p* = 0.037	0.52	1.58 (0.87, 2.87), *p* = 0.131	1.1 (0.56, 2.16), *p* = 0.775	1.48 (0.89, 2.44), *p* = 0.13	0.93 (0.41, 2.13), *p* = 0.873	1.73 (0.74, 4.02), *p* = 0.203	1.16 (0.73, 1.83), *p* = 0.533
**Short-term end*points***										
**30-day all-cause mortality**	45 (9.1%)	19 (11.4%)	1.63 (0.83 to 3.21), *p* = 0.158	0.52	2.19 (0.92, 5.21), *p* = 0.076	1.21 (0.41, 3.58), *p* = .737	1.85 (0.88, 3.92), *p* = 0.106	1.19 (0.31, 4.57), *p* = 0.805	3.33 (1.02, 10.86), *p* = 0.046	1.12 (0.54, 2.32), *p* = 0.762
**Rehospitalisation within 30 days**	53 (10.7%)	19 (11.4%)	1.22 (0.65 to 2.29), *p* = 0.534	0.51	0.79 (0.33, 1.88), *p* = 0.589	1.33 (0.53, 3.37), *p* = 0.545	0.98 (0.47, 2.06), *p* = 0.967	1.2 (0.41, 3.5), *p* = 0.743	0.98 (0.23, 4.19), *p* = 0.98	1 (0.54, 1.87), *p* = 0.99
**Mean length of stay, days (SD)**	10.0 (6.9)	10.1 (7.5)	-0.23 (-1.62 to 1.16), *p* = 0.743	-	0.28 (-1.7, 2.27), *p* = 0.779	0.26 (-1.65, 2.17), *p* = 0.789	1.02 (-0.64, 2.68), *p* = 0.229	-1.72 (-3.89, 0.45), *p* = 0.119	-0.2 (-2.67, 2.28), *p* = 0.876	0.32 (-1.16, 1.81), *p* = 0.67
**Decline Barthel's Index score (points) after 30 days**	68 (13.7%)	29 (17.4%)	1.5 (0.85 to 2.66), *p* = 0.161	0.53	2.02 (0.96, 4.26), *p* = 0.065	1.47 (0.61, 3.54), *p* = 0.393	1.67 (0.88, 3.18), *p* = 0.117	1.47 (0.53, 4.07), *p* = 0.463	3.6 (1.33, 9.78), *p* = 0.012	1.07 (0.58, 2), *p* = 0.823
**Long-term endpoint**										
**180-day all-cause mortality**	147 (29.6%)	47 (28.1%)	0.82 (0.5 to 1.35), *p* = 0.445	0.49	1.03 (0.56, 1.89), *p* = 0.929	1.22 (0.6, 2.47), *p* = 0.579	1.09 (0.65, 1.83), *p* = 0.745	0.88 (0.35, 2.21), *p* = 0.79	0.73 (0.32, 1.68), *p* = 0.457	1.06 (0.66, 1.7), *p* = 0.816

Abbreviations: SD denotes standard deviation; OR denotes odds ration; AUC denotes area under the curve; BMI denotes body mass index; NRS denotes nutritional risk screening 2002

P values were adjusted for important confounders including age, BMI, nutritional support intervention, contributing center, presence of stroke, COPD, hypertension, diabetes, chronic heart failure

Supplementary Table S3. Effects of nutritional support on clinical outcomes of patients in the lowest and in the other three quartiles of SMI at level L3

	high SMI			low SMI			
	**Control group (*n* = 177)**	**Intervention group (*n* = 211)**	**OR or Coefficient (95% CI), p value adjusted**	**Control group (*n* = 67)**	**Intervention group (*n* = 64)**	**OR or Coefficient (95% CI), *p* value adjusted**	***p* for interaction**
**Primary endpoint**							
Adverse clinical outcome within 30 days	55 (31.1%)	58 (27.5%)	0.84 (0.54, 1.30), *p* = 0.439	24 (36%)	15 (23%)	0.55 (0.26, 1.18), *p* = 0.123	0.65
**Short-term endpoints**							
30-day all-cause mortality	20 (11.3%)	16 (7.6%)	0.64 (0.32, 1.28), *p* = 0.212	10 (15%)	6 (9%)	0.59 (0.20, 1.73), *p* = 0.336	0.91
Rehospitalisation within 30 days	21 (11.9%)	27 (12.8%)	1.09 (0.59, 2.00), *p* = 0.781	7 (10%)	6 (9%)	0.89 (0.28, 2.79), *p* = 0.837	0.81
Length of hospital stay	10.1 (6.5)	10.1 (7.5)	0.48 (-1.37, 1.46), *p* = 0.947	11.1 (7.6)	9.0 (6.4)	-2.08 (-4.52, 0.36), *p* = 0.094	
Decline Barthel index Score	35 (18.8%)	24 (11.4%)	0.52 (0.29, 0.91), *p* = 0.023	15 (22%)	5 (8%)	0.29 (0.09, 0.86), *p* = 0.026	0.56
**Long-term endpoints**							
180-day all-cause mortality	58 (32.8%)	68 (32.2%)	0.97 (0.63, 1.49), *p* = 0.910	25 (37%)	15 (23%)	0.51 (0.24, 1.10), *p* = 0.087	0.52

Abbreviations: OR denotes odds ratio; CI denotes Confidence Interval

P values were adjusted for important confounders including age, BMI, nutritional support intervention, contributing center, presence of stroke, COPD, hypertension, diabetes, chronic heart failure
